# Comparison of effect of auriculotherapy and mefenamic acid on the severity and systemic symptoms of primary dysmenorrhea: a randomized clinical trial

**DOI:** 10.1186/s13063-021-05622-w

**Published:** 2021-09-26

**Authors:** Masoomeh Vahedi, Seyedeh Batool Hasanpoor-Azghady, Leila Amiri-Farahani, Imaneh Khaki

**Affiliations:** Department of Midwifery and Reproductive, Nursing Care Research Center (NCRC), School of Nursing and Midwifery, Iran University of Medical Sciences, Rashid Yasemi st., Valiasr St, Tehran, 1996713883 Iran

**Keywords:** Primary dysmenorrhea, Auriculotherapy, Mefenamic acid, Systemic symptoms

## Abstract

**Background:**

Primary dysmenorrhea (PD) is the most common complaint in young women and adolescents. Side effects of non-steroidal anti-inflammatory drugs can limit their use. Therefore, non-pharmacological pain relief methods such as auriculotherapy may play an important role in PD management. This study was conducted to compare the effect of auriculotherapy and mefenamic acid on the severity and systemic symptoms of PD.

**Methods:**

In a randomized clinical trial, 83 students were randomized into two groups. In the auriculotherapy group, electrical stimulation of the ear was conducted once a week for two menstrual cycles. In each cycle close to menstruation, ear seeds were inserted on pressure points to be pressed in times of pain. In the mefenamic acid group, subjects took mefenamic acid capsules upon seeing the initial symptoms of menstruation until the pain reduces. The primary outcomes were mean pain intensity and systemic symptoms associated with it. Pain intensity was measured through the visual analog scale (VAS) and the verbal multidimensional scoring system (VMS). Systemic symptoms were assessed using VMS, as well as the yes/no question form.

**Results:**

Mean pain intensity with the VAS was significantly lower in the auriculotherapy group than the mefenamic acid group in the first and second cycles of intervention. There was a significant difference in VMS grade between both groups during the second cycle of intervention. In terms of the systemic symptoms in the second cycle of intervention, no subjects had dysmenorrhea grade 3 (common systemic symptoms) in the auriculotherapy group. Whereas in the mefenamic acid group, 16.7% of the subjects still had dysmenorrhea grade 3. There was no significant difference between the two groups in the frequency of systemic symptoms of PD. There was a significant decrease in the frequency of fatigue and diarrhea in both groups. However, there was a significant reduction in the frequency of nausea, headache, and anger in the auriculotherapy group.

**Conclusion:**

Mean pain intensity with the VAS was lower with the auriculotherapy. Also, 65.9% of auriculotherapy group subjects were in the dysmenorrhea grades 0 and 1. Therefore, auriculotherapy is recommended because of its fewer complications and more effect on PD.

**Trial registration:**

ClinicalTrials.gov IRCT20181207041873N1. Registered on February 24, 2019. https://en.irct.ir/user/trial/35967/view

## Background

Primary dysmenorrhea (PD) begins at the same time or before menstrual bleeding and usually lasts for 2 or 3 days [1]. The mechanism of pain will result in the increased production of prostaglandins. PD may be accompanied by systemic symptoms such as headache, dizziness, nausea, vomiting, diarrhea, and fatigue [2]. The prevalence of PD has been reported to be 16–81% [3]. Various studies in Iran have reported the PD prevalence between 74 and 90% [4]. PD can reduce mental focus in the classroom, limit social activities, reduce educational achievement, increase absenteeism from school or workplace, and reduce the quality of life [5].

Non-steroidal anti-inflammatory drugs (NSAIDs) and oral contraceptives are used as first-line treatment of PD [6, 7]. NSAIDs reduce menstrual pain by 20 to 25% in women [8] through inhibiting prostaglandin synthesis [2]. NSAID consumption is associated with side effects including gastrointestinal disorders, nephrotoxic problems, blood disorders, headaches, and drowsiness. Scientific evidence suggests a positive effect of alternative medicine interventions in managing dysmenorrhea symptoms which could be a therapeutic approach [9, 10]. Furthermore, as healthcare costs increase in countries, the use of non-pharmacological treatments, including traditional and complementary therapies is becoming increasingly important [11].

Auriculotherapy, as the Traditional Chinese Medicine (TCM), treats illness through stimulation of pressure points using needles, electric current, laser, heat, and seed (Vaccaria plant seed, magnetic beads) [12]. Ear stimulation can inhibit prostaglandin overproduction, reduce cerebral cortical excitability, and regulate hormone secretion from endocrine glands [13]. According to the traditional Chinese medicine theories, PD and its associated symptoms are caused by energy deficiency or stagnation in the uterus, and the treatment for PD is the modulation of the flow of energy and the blood, and the regulation of the organs of the body, particularly the liver, spleen, and kidneys [12]. Auriculotherapy improves organ function and balances the blood flow [10]. Auriculotherapy rarely has any side effects. The most common side effects of auriculotherapy are that the ear may become red and tender after treatment, but this redness and tenderness are temporary. Due to endorphin release, some patients may even experience drowsiness and dizziness and should lie down for a while [12].

The results of a systematic review study indicated that women who received acupuncture on the body or ears had a greater reduction in the severity of primary dysmenorrhea and systemic symptoms than women who received NSAIDs. Although the study noted that the overall quality of the studies was very low, the scales used to assess symptoms were unknown or unclear [13].. The results of another systematic review also showed that the clinical effectiveness of ear or body acupuncture was higher than NSAIDs except in two studies [14]. Despite all the evidence, the difference in the applied acupuncture style, the reported and follow-up results, and the risk of bias, further studies need to be conducted on the value of acupuncture in the treatment of PD and providing a more definitive result [15]. The researchers did not find a systematic review study with the sole focus on the effect of auriculotherapy in PD. However, various studies in this field have shown that auriculotherapy reduces menstrual cramps and improves the quality of life in people with PD [16, 17]. However, studies that investigated the effect of auriculotherapy on PD either lacked the common medical treatment or had no baseline cycle and intervention for two consecutive cycles. Currently, mefenamic acid is widely used to reduce primary dysmenorrhea [18]. Due to the expansion of the science of auriculotherapy, this study aimed to compare the effect of auriculotherapy and mefenamic acid on the severity and systemic symptoms of primary dysmenorrhea.

## Methods

### Study design

This study is a randomized clinical trial with two parallel groups that was conducted on students of Qom University of Medical Sciences (Qom, Iran) between March and July 2019, which included three evaluations in the control cycle (baseline) and two intervention cycles. The Medical Research Ethics Committee of Iran University of Medical Sciences approved this study with code (IR.IUMS.REC.1397.550). From all subjects, written consent was freely obtained. The study was registered at the Iranian Registry of Clinical Trials (IRCT) with code number 20181207041873N1. The present study adheres to the Consolidated Standards of Reporting Trials (CONSORT) guidelines.

### Sample size estimation

The sample size calculation was conducted using a similar study [11] with *α* = 0.05 and *β* = 0.2. Considering the minimum clinical difference of 0.3 scores in the mean score of pain intensity with VAS between the two groups that showed a statistically significant difference, the sample size of 28 people were estimated for each group. Besides, the sample size was calculated according to the VMS, which measures both pain intensity and systemic symptoms of primary dysmenorrhea. Considering the minimum 30% clinical differences in terms of frequency in VMS grades between the two groups that showed a statistically significant difference, the sample size of 38 people were estimated for each group. Since the sample size obtained based on VMS was higher, this variable was used to determine the sample size. However, 45 people were assigned to each group, taking into account 20% sample drop.

### Participants

**Fig. 1:**
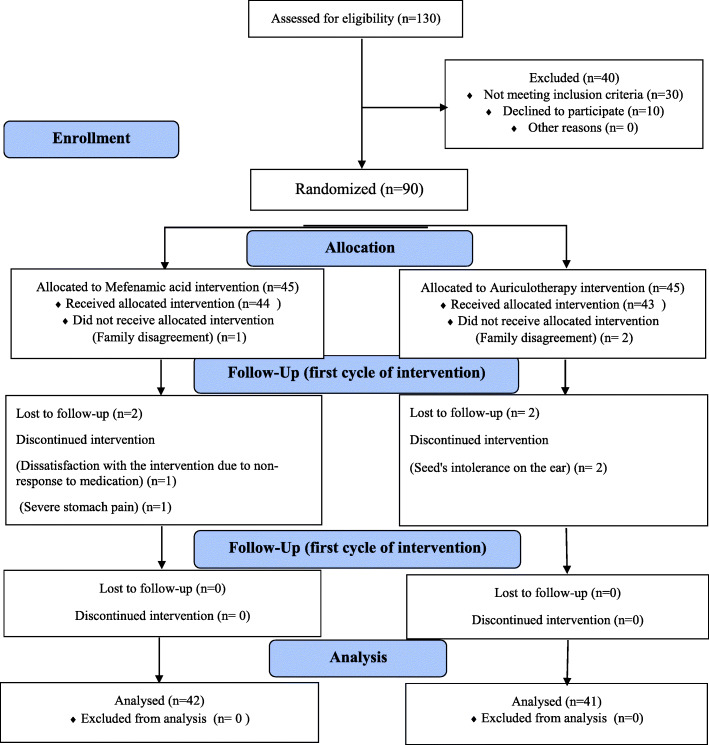
Enrolment of participants into two groups of mefenamic acid and auriculotherapy

During a recall by the Qom University of Medical Sciences, some information on the objectives and method of the study, the confidentiality of information, withdrawing from the study at any time participants wish were explained to students. Participants who were willing to participate in the study introduced themselves by telephone. At the time of introduction, if participants had any questions, all of their questions would be answered in full so that they would be fully aware of the intervention method and the duration of participation in the study. In this way, the researchers tried to minimize samples drop. A total of 130 samples were screened which 30 people lacked the inclusion criteria, while ten people after having fully informed on the intervention methods were reluctant to participate in the study. The enrollment was done by a researcher who later performed the intervention in two groups (Fig. 1).

### Eligibility criteria

#### The inclusion criteria

The following were the inclusion criteria:

- Being single
- Having Iranian nationality
- Age range 18–35 years
- Having regular menstrual cycles (the period length duration of 3–7 days with an interval of 24–32 days between the last two menstrual cycles)
- PD grades 2 and 3 according to VMS (this inclusion criterion was not revealed to participants to avoid bias), the experience of PD in most menstrual cycles.

#### The exclusion criteria

The following were the exclusion criteria:

- Not being diagnosed with any known chronic disease
- Not having a heart pacemaker
- Not having any anemia or weakness history
- Not taking OCP in the last 3 months
- Not proceeding to lose weight during the study
- Lack of regular physical activity 3 months before the beginning of the study
- Not having a body mass index over 30 kg/m^2^
- Not consuming tobacco products (cigarettes, hookahs, and drugs) and alcohol
- Having no history of abdominal and pelvic surgery or an abnormal uterine ultrasound, lack of any severe psychological stress in the last 6 months
- Having no mass, swelling, and scarring on the external surface of both ears in the auriculotherapy group
- Having no history of auriculotherapy in the past 6 months

#### The withdrawal criteria

The following were the withdrawal criteria:

- The diagnosis of gynecological disease during the study by a specialist physician
- Unpleasant stressful events during the study
- Failure to attend all auriculotherapy sessions
- Not applying pressure on the embedded ear seeds despite researchers follow-ups at least four times a day.

### Randomization, concealment of allocation, and blinding

During the sampling process, enrolled students were randomly assigned to two groups at a ratio of 1:1. The randomization sequence is generated by computers with a four-block size. A statistician who was not present in the study made a randomization list. For allocation concealment, the assignment list remained with this statistician. The researcher conducting the intervention, after receiving written informed consent and freely form via phone or SMS, understood that every subject should be in which group. This researcher did not know anything about the random allocation technique. Eligible students were randomized into two groups: auriculotherapy and mefenamic acid until the completion of the sampling. Blinding was not possible due to the nature of interventions.

### Intervention

Individuals in both auriculotherapy and mefenamic acid groups were initially evaluated in a control cycle. There was no intervention in this cycle, and all people only completed the menstrual status baseline information form. Then in another two cycles, interventions were performed for each group. In terms of research ethics, we could not forbid our samples from taking painkillers or any other action when the pain became unbearable in two cycles of intervention. Therefore, if any of the samples on either group feel the need to take some painkillers, except for the intervention of the same group, painkiller consumption or any other common action was allowed, except for the use of mefenamic acid for relief. When the pain was tolerable, the samples were asked to discontinue painkiller consumption or any other common action. In the case of taking painkillers, before consumption, participants were expected to assign a number to their pain intensity on the VAS and write down the type of drug they use or any similar action for that matter.

### Auriculotherapy group

In this group, auriculotherapy was performed by one of the researchers of the present study. Auriculotherapy is performed through electrical stimulation of the ear. Each ear was sterilized with 70% alcohol and a minute of relaxing massage using Pointer Excel II stimulator at the frequency of 2 Hz and current intensity of 2–4 mA applied for 20 s on each point. The procedure was performed once a week for two menstrual cycles per person. Eleven points were electrically stimulated. The main points include the Shen Men, thalamus, zero, and endocrine, and anatomic points include the uterus, internal genitalia, ovary, and the supporting points include the kidney, pelvis, vagus nerve, and prostaglandin. To prevent the samples drop, the researcher was available on the day of the auriculotherapy whenever the samples had free time. At the last session of electrical stimulation, which was close to menstruation, four points of the Shen Men, thalamus, uterus, and pelvis are planted with vaccaria seeds from the vaccaria plant made by Huan Qiu Company in China and stabilized on the external surface of both ears using matte glue. Then participants were asked to apply pressure on seeds at the onset of menstruation pain. Each point was pressed 4–6 times daily for 1 min, with at least 1 h in between. Pressing the points continued each day until menstrual pain reduced. The force of the seeds was enough to cause pain and a burning sensation in the external ear. Participants were explained that they can experience various sensations on pressure points, including numbness, swelling, mild pain, or heat. At the same time, the given explanation was once again sent via SMS to group auriculotherapy. Seeds were then removed 24 h after pain relief from menstrual pain. At this time, the number, duration, and amount of pressing of the seeds were checked by the researcher via phone and SMS on social media. Samples were asked to report by phone and SMS on social media if they encountered any problems that caused the seeds not to be pressed as described.

### Mefenamic acid group

The mefenamic acid group received mefenamic acid capsules (250 mg capsules from Amin pharmaceutical company) during two menstrual cycles and upon seeing the first signs of menstrual onset such as cramp, pain, and bleeding. They took two capsules for the first time then, proceeded to take one capsule every 6 h, and continued doing this until pain reduced.

### Outcome measures

#### Primary outcome measure

The primary outcomes were mean pain intensity and systemic symptoms associated with it. Pain intensity was measured through the visual analog scale (VAS) and the verbal multidimensional scoring system (VMS). Systemic symptoms were assessed using VMS, as well as the yes/no question form. Systemic symptoms were headache, fatigue, nausea, vomiting, nervousness, and diarrhea during menstruation.

We used two scales to measure pain intensity. We used VAS to compare the results of our study with other studies that have mostly used this scale to measure the severity of pain in PD. On the other hand, VMS as a multidimensional tool may be more accurate measures the grade of pain in PD.

VAS consist of a 10-cm horizontal line grading from zero to 10. Zero typically represents “no pain at all,” whereas 10 signifies “worst pain” imaginable. Samples were asked to record the most severe menstrual pain they felt on the VAS within the first 3 days of menstruation during the control cycle and the first and second treatment cycle on a data entry form. VAS is one of the most commonly used measures of pain intensity in pain research, which has been repeatedly proven to be reliable in various studies [19, 20].

The VMS grading system ranges from grades 0 to 3 for evaluating the working ability and the systemic symptoms, and whether analgesia is required or not (Table 1).

**Table 1:** The verbal multidimensional scoring system (VMS)

Grade	Working ability	Systemic symptoms	Analgesia
Grade 0: Menstruation is not painful and daily activity is unaffected	Unaffected	None	Not required
Grade 1: Menstruation is painful but seldom inhibits the woman’s normal activity. Analgesics are seldom required. Mild pain	Rarely affected	None	Rarely required
Grade 2: Daily activity affected. Analgesics required and give relief so that absence from work or school is unusual. Moderate pain	Moderately affected	Few	Required
Grade 3: Activity clearly inhibited. Poor effect of analgesics. Vegetative symptoms, e.g. headache, tiredness, nausea, vomiting, and diarrhea. Severe pain	Clearly inhibited	Apparent	Poor effect

VMS has been used in various studies. Its reliability and validity have been proven in different studies [19, 20]. Subjects were trained that according to Table 1, they should record the degree of their most severe menstrual pain within the first 3 days of menstruation in the control cycle and the first and second treatment cycle.

### Adverse events

Auriculotherapy rarely has any side effects. The most common side effect is that the ear may become red and tender after treatment, but it is temporary redness and tenderness. Due to endorphin release, some patients may even experience drowsiness and dizziness and should lie down for a while [12]. NSAIDs such as mefenamic acid are used as the first-line treatment of PD [6, 7]. NSAID consumption is associated with side effects including gastrointestinal disorders, nephrotoxic problems, blood disorders, skin reactions, headaches, and drowsiness.

Potential adverse events (AEs) of auriculotherapy and mefenamic acid consumption were explained to all study participants before signing the informed consent. AEs experienced by the subjects at any point in the trial were reported to the investigators and recorded on the case report form (CRF). In addition to sample self-report, the researcher in charge of sampling sought all participants about any AEs that might occur during the intervention period. The frequency of adverse events (AEs) was recorded in two intervention cycles.

### Satisfaction rate

The frequency of satisfaction with each of the two intervention methods of auriculotherapy and mefenamic acid consumption was recorded in two intervention cycles.

### Data collection and management

Demographic and menstrual status data were obtained at visit one by a general information questionnaire. The questionnaire consisted of two parts: the first part was demographic information including age, education, height, weight, and living in the dorm. The second part was menstrual status information including menarche age, age of onset of dysmenorrhea, menstrual intervals, duration of menstruation, and amount of menstrual bleeding. Eligible samples were asked to record their dysmenorrhea diary in one control cycle and two intervention cycles. All of the primary outcome measures and adverse events (AEs) were obtained from the dysmenorrhea diary. The dysmenorrhea diary was included VAS, VMS, painkiller consumption, satisfaction with the intervention method, AEs, and a questionnaire that assessed the frequency of systemic symptoms with two yes and no answers. Systemic symptoms were headache, fatigue, nausea, vomiting, nervousness, and diarrhea during menstruation. Data recorded by the samples were collected after each menstrual cycle. All paper-based data were checked by the investigators. Then, data managers used Microsoft Excel 2016 software to input data and establish an electronic database. They have also used password protection. A data manager will check all the data without knowing the treatment allocation. All researchers had access to the final database. To ensure the confidentiality of the data, all subjects were determined with numbers.

### Quality control and monitoring

To guarantee the quality of the study, auriculotherapy was performed by one of the researchers of the present study who had been trained by a person with more than 10 years of experience in auriculotherapy and acupuncture and had been working in auriculotherapy for more than 6 months. An expert in the field of auriculotherapy and acupuncture was conducted regular monitoring to ensure the integrity and authenticity of all data. During these visits, she verified all consent forms, complied with established protocol, and also wrote a monitoring report after each visit.

All samples were taught how according to the verbal multidimensional scoring system (VMS), and VMS record the severity of their most severe menstrual pain in the first 3 days of menstruation in the control cycle and first and second cycles of treatment.

### Statistical methods

Data analysis was performed using SPSS software version 26. In the inferential part, chi-square or Fisher’s exact test was used to compare qualitative variables, while an independent *t* test and univariate analysis of covariance (ANCOVA) were used to compare quantitative variables in two groups. Analysis of variance with repeated measures was used for the comparison of the mean pain intensity over time (control cycle and the first and second treatment cycle). Friedman’s test was applied to compare VMS frequency over time (three cycles), while Cochrane’s *Q* test was used to compare systemic symptoms frequency over time (three cycles). Bonferroni test was used for making the two-by-two comparisons of mean intensity, while for pairwise comparison of VMS ranked variables, the Wilcoxon test was used. ANCOVA was used to control the effect of painkiller consumption on pain intensity. The Mantel-Haenszel test was used to control the effect of painkiller consumption on systemic symptoms associated with primary dysmenorrhea. In all of these tests, the significance level of less than 0.05 was considered.

## Results

From 45 participants in each group, in the auriculotherapy group, two individuals due to family’s disagreement to proceed with the intervention and two individuals for seed’s intolerance on the ear were excluded from the study. While in the mefenamic acid group, one individual for family’s disagreement to proceed with the intervention, one individual for severe stomach pain while taking medication, and one individual due to lack of response to treatment were excluded from the study. Therefore, the auriculotherapy group consists of 41 subjects, while the mefenamic acid group comprised 42 subjects (Fig. 1). There was no statistically significant difference between the two groups in terms of demographic variables and menstrual cycle characteristics, they were homogeneous. In terms of painkiller consumption, the two groups had a statistically significant difference in the two intervention cycles. The two groups used only painkillers in three cycles: control, the first, and the second intervention cycles and did not do any other action to reduce pain. Demographic and menstrual cycle characteristics are outlined in Table 2.

**Table 2:** Comparison of demographic and menstruation cycles’ characteristics of the subjects

Variables	Auriculotherapy group (***n*** = 41)	Mefenamic acid group (***n*** = 42)	***P*** value
^a^Age (year, mean ± SD^b^)	22.66 ± 2.73	22.74 ± 3.08	0.904
^c^Education, *n* (%)			
Bachelor	30 (73.2)	31 (73.8)	0.994
Masters	6 (14.6)	6 (14.3)	
Doctorate	5 (12.2)	5 (11.9)	
^b^BMI (kg/m^2^, mean ± SD)	22.69 ± 2.38	22.83 ± 2.81	0.796
^b^Age of menarche (year, mean ± SD)	12.68 ± 1.45	12.83 ± 1.56	0.653
^b^Age of onset of dysmenorrhea (year, mean ± SD)	14.07 ± 1.83	13.93 ± 1.67	0.706
^b^Menstrual interval (day, mean ± SD)	28.8 ± 2.41	29 ± 2.25	0.74
^b^Menstrual duration (day, mean ± SD)	6.2 ± 0.87	6.12 ± 0.99	0.713
^c^Amount of menstrual bleeding, *n* (%)			
Mild	7 (17.1)	6 (14.3)	0.922
Moderate	28 (68.3)	29 (69)	
Severe	6 (14.6)	7 (16.7)	
^c^Living in dorms, *n*(%)			
Yes	28 (68.3)	28 (66.7)	0.871
No	13 (31.7)	14 (33.3)	
^d^Painkiller consumption: control cycle, *n*(%)			
Yes	38 (92.7)	40 (95.2)	0.625
No	3 (7.3)	2 (4.8)	
^c^Painkiller consumption: 1st cycle, *n*(%)			
Yes	27 (65.9)	15 (35.7)	0.006
No	14 (34.1)	27 (64.3)	
^c^Painkiller consumption: 2nd cycle, *n*(%)			
Yes	27 (65.9)	15 (35.7)	0.006
No	14 (34.1)	27 (64.3)	

^a^Independent *t* test, ^b^Standard deviation, ^c^chi-square test, and ^d^Fisher’s exact test

**Fig. 2:**
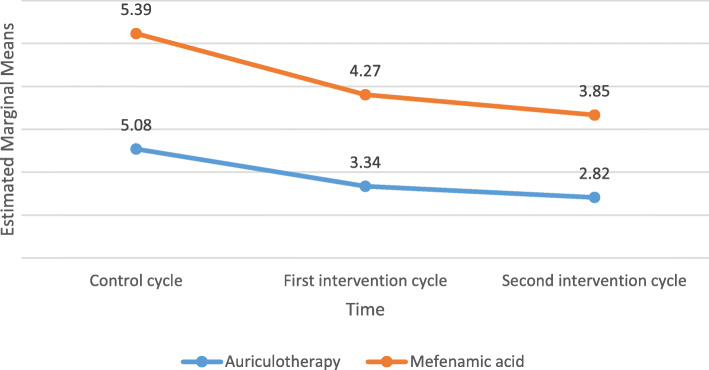
Average scores of the maximum level of pain severity during the first three days of menstruation in the control cycle, the first intervention cycle, and the second intervention cycle

In between-group comparison, there was no statistically significant difference in the mean score of pain intensity between both groups in the control cycle, but this mean was significantly lower in the first and second intervention cycles of the auriculotherapy group compared to the mefenamic acid group (Fig. 2). The results from the within-group comparison indicated that there was a significant difference between the three measurement times in the auriculotherapy group. Bonferroni’s two-by-two comparison indicated that the mean score of pain intensity in the auriculotherapy in the first intervention cycle (mean difference: −1.74; 95 CI −2.19/−1.28; *P* <0.001 ) and second intervention cycle (mean difference: −2.26; 95 CI −2.74/−1.77; *P* <0.001 ) was significantly lower than the control cycle. Also, the mean pain intensity score of the second intervention cycle was significantly lower than the first intervention cycle (mean difference: −0.52; 95 CI −.94/ −.09; *P* <0. 012 ).

In the mefenamic acid group, the results of Bonferroni’s two-by-two comparison indicated that there was a statistically significant difference in the mean pain intensity score in the first intervention cycle (mean difference: −1.11; 95 CI −1.73/ −0.5; *P* <0.001 ) and the second intervention cycle (mean difference: −1.54; 95 CI −2.16/ −0.91; *P* <0.001 ) compared to the control cycle, it was lower than the control cycle (Table 3).

**Table 3:** The severity of dysmenorrhea assessed by the VAS in two groups of study in the control cycle, the first intervention, and the second intervention cycle

Time	Auriculotherapy group (***n*** = 41)		Mefenamic acid group (***n*** = 42)		Unadjusted ***P*** value (between groups)	Adjusted ***P*** value (between groups)	^f^ES (between)
	Mean ± SD^a^	^b^Adjusted mean (95% CI)	mean ± SD	^b^Adjusted mean (95% CI)			
^c^Control cycle	5.08 ± 1.74	-	5.39 ± 1.73		0.423	-	-
^d^1st cycle	3.34 ± 1.81	3 (2.42 to 3.58)	4.27 ± 2.33	4.59 (4.02 to 5.16)	0.046	< 0.001	0.15
^d^2nd cycle	2.82 ± 1.77	2.63 (2.06 to 3.21)	3.85 ± 1.98	4.03 (3.46 to 4.59)	0.015	0.001	0.12
^e^*P* value (within groups)	< 0.001		< 0.001				

^a^Standard deviation, ^b^adjusted mean using analysis of covariance after controlling for painkiller consumption variable, ^c^independent *t* test, ^d^univariate ANCOVA, ^e^repeated measures ANOVA, and ^f^effect size (ES) based on partial eta square

Table 4 shows the difference between the mean pain intensities of menstrual cycles.

**Table 4:** Comparison of the difference in VAS scores between groups

Time	Auriculotherapy group (***n*** = 41)	Mefenamic acid group (***n*** = 42)	^**a**^***P*** value	95% CI (L/H)
	Mean ± SD^b^	Mean ± SD		
1st cycle–control cycle	−1.73 ± 1.17	−1.11 ± 1.58	0.047	−1.2/−0.008
2nd cycle–control cycle	−2.26 ± 1.25	−1.53 ± 1.63	0.027	−1.3/−0.08
2nd cycle–1st cycle	−0.52 ± 1.09	−0.42 ± 1.09	0.68	−0.57/0.37

^a^Independent *t* test and ^b^standard deviation

The between-group comparison showed no statistically significant difference in terms of VMS grade between both groups during the control cycle and the first intervention cycle. However, both groups showed a statistically significant difference in the second intervention cycle in such a way that in the auriculotherapy group, we did not have any subject suffering from dysmenorrhea grade 3 in the second cycle. 65.9% of subjects had grade zero to one, whereas in the mefenamic acid group, 16.7% of samples were still suffering from dysmenorrhea grade 3, and only 23.8% had grade zero to one.

In within-group comparison in the auriculotherapy group, the results of the two-by-two comparison based on the Wilcoxon test indicated that the VMS of the control cycle was significantly different than the first intervention cycle (*P*<0.001) and the second intervention cycle (*P*<0.001), and the first treatment cycle with the second treatment cycle (*P*= 0.011). Besides, the results of the Wilcoxon two-by-two comparison in the mefenamic acid group indicated that the VMS of the control cycle was significantly different from the first intervention cycle (*P* = 0.001) and the second intervention cycle (*P* = 0.007) (Table 5).

**Table 5:** Severity of dysmenorrhea assessed by VMS in two groups of study on the first day of the control cycle, the first intervention cycle, and the second intervention cycle

Time	Grade	Auriculotherapy group (***n*** = 41) ***n***(%)	Mefenamic acid group (***n*** = 42) ***n***(%)	***P*** value (between groups)
^a^Control cycle	2	31 (75.6)	32 (76.2)	0.95
	3	10 (24.4)	10 (23.8)	
^b^1st cycle	0	5 (12.2)	4 (9.5)	0.06
	1	13 (31.7)	10 (23.8)	
	2	21 (51.2)	17 (40.5)	
	3	2 (4.9)	11 (26.2)	
^b^2nd cycle	0	7 (17.1)	2 (4.8)	<0.001
	1	20 (48.8)	8 (19)	
	2	14 (34.1)	25 (59.5)	
	3	0 (0)	7 (16.7)	
^c^*P* value (within groups)		< 0.001		0.003

^a^Chi-square test, ^b^Fisher’s exact test, and ^c^Friedman test

There was no significant difference between the two groups in the frequency of systemic symptoms of primary dysmenorrhea (Table 6).

**Table 6:** Comparison of systemic symptom reported during menstruation between groups

Time	Painkiller consumption	Systemic symptom		Auriculotherapy group (***n*** = 41) ***n***(%)	Mefenamic acid group (***n*** = 42) ***n***(%)	***P*** value	^**c**^Adjusted ***P*** value
Control cycle	-	Nausea	Yes	22 (53.7)	15 (35.7)	^a^0.1	-
			No	19 (46.3)	27 (64.3)		
1st cycle	Yes	Nausea	Yes	10 (37)	10 (66.7)	^a^0.065	0.388
			No	17 (63)	5 (33.3)		
	No	Nausea	Yes	4 (28.6)	6 (22.2)	^b^0.712	
			No	10 (71.4)	21 (77.8)		
2nd cycle	Yes	Nausea	Yes	4 (14.8)	5 (33.3)	^b^0.242	0.467
			No	23 (85.2)	10 (66.7)		
	No	Nausea	Yes	4 (28.6)	8 (29.6)	^b^1.000	
			No	10 (71.4)	19 (70.4)		
Control cycle	-	vomiting	Yes	4 (9.8)	6 (14.3)	^b^0.73	-
			No	37 (90.2)	36 (85.7)		
1st cycle	Yes	vomiting	Yes	3 (11.1)	3 (20)	^b^0.649	0.745
			No	24 (88.9)	12 (80)		
	No	vomiting	Yes	0	0	-	
			No	14 (100)	27 (100)		
2nd cycle	Yes	vomiting	Yes	0 (0)	1 (6.7)	^b^0.357	0.831
			No	27 (100)	14 (93.3)		
	No	vomiting	Yes	1 (7.1)	1 (3.7)	^b^1.000	
			No	13 (92.9)	26 (96.3)		
Control cycle	-	Headache	Yes	16 (39)	18 (42.9)	^a^0.72	
			No	25 (61)	24 (57.1)		
1st cycle	Yes	Headache	Yes	12 (44.4)	8 (53.3)	^a^0.580	0.949
			No	15 (55.6)	7 (46.7)		
	No	Headache	Yes	5 (35.7)	9 (33.3)	^b^1	
			No	9 (64.3)	18 (66.7)		
2nd cycle	Yes	Headache	Yes	4 (14.8)	6 (40)	^b^0.128	0.065
			No	23 (85.2)	9 (60)		
	No	Headache	Yes	4 (28.6)	13 (48.1)	^a^0.228	
			No	10 (71.4)	14 (51.9)		
Control cycle	-	Fatigue	Yes	36 (87.8)	37 (88.1)	^b^0.999	-
			No	5 (12.2)	5 (11.9)		
1st cycle	Yes	Fatigue	Yes	21 (77.8)	11 (73.3)	^b^1	0.999
			No	6 (22.2)	4 (26.7)		
	No	Fatigue	Yes	11 (78.6)	21 (77.8)	^b^1	
			No	3 (21.4)	6 (22.2)		
2nd cycle	Yes	Fatigue	Yes	16 (59.3)	11 (73.3)	^a^0.362	0.960
			No	11 (40.7)	4 26.7)		
	No	Fatigue	Yes	11 (78.6)	19 (70.4)	^b^0.719	
			No	3 (21.4)	8 (29.6)		
Control cycle	-	Diarrhea	Yes	18(43.9)	12(28.6)	^a^0.14	-
			No	23 (56.1)	30 (71.4)		
1st cycle	Yes	Diarrhea	Yes	5 (18.5)	4 (26.7)	^b^0.698	0.852
			No	22 (81.5)	11 (73.3)		
	No	Diarrhea	Yes	3 (21.4)	4 (14.8)	^b^0. 673	
			No	11 (78.6)	23 (85.2)		
2nd cycle	Yes	Diarrhea	Yes	3 (11.1)	2 (13.3)	^b^1.000	0.831
			No	24 (88.9)	13 (86.7)		
	No	Diarrhea	Yes	4 (28.6)	4 (14.8)	^b^0.411	
			No	10 (71.4)	23 (85.2)		
Control cycle	-	Nervousness	Yes	28 (68.3)	27 (64.3)	^a^0.69	-
			No	13 (31.7)	15 (35.7)		
1st cycle	Yes	Nervousness	Yes	13 (48.1)	7 (46.7)	^a^0.927	0.692
			No	14 (51.9)	8 (53.3)		
	No	Nervousness	Yes	6 (42.9)	16 (59.3)	^a^0.318	
			No	8 (57.1)	11 (40.7)		
2nd cycle	Yes	Nervousness	Yes	10 (37)	5 (33.3)	^a^0.810	0.221
			No	17 (63)	10 (66.7)		
	No	Nervousness	Yes	3 (21.4)	16 (59.3)	^a^0.021	
			No	11 (78.6)	11 (40.7)		

^a^Chi-square test, ^b^Fisher’s exact test, and ^c^adjusted *P* value using Mantel-Haenszel after controlling for painkiller consumption variable

In both groups, there was a significant decrease in the frequency of fatigue and diarrhea, although there was a significant decrease in symptoms of nausea, headache, and anger in the auriculotherapy group (Table 7).

**Table 7:** Comparison of systemic symptom reported during menstruation within-group

Systemic symptom	Time	Auriculotherapy group (***n*** = 41) ***n*** (%)	Mefenamic acid group (***n*** = 42) ***n*** (%)	Systemic symptom	Time	Auriculotherapy group (***n*** = 41) ***n*** (%)	Mefenamic acid group (***n*** = 42) ***n*** (%)
Nausea	Control cycle	22 (53.7)	15 (35.7)	Fatigue	Control cycle	36 (87.8)	37 (88.1)
	1st cycle	14 (34.1)	16 (38.1)		1st cycle	32 (78)	32 (76.2)
	2nd cycle	8 (19.5)	13 (31)		2nd cycle	27 (65.9)	30 (71.4)
	^a^*P* value	0.002	0.58		^a^P value	0.02	0.02
Vomiting	Control cycle	4 (9.8)	6 (14.3)	Diarrhea	Control cycle	18 (43.9)	12 (28.6)
	1st cycle	3 (7.3)	3 (7.1)		1st cycle	8 (19.5)	8 (19)
	2nd cycle	1 (2.4)	2 (4.8)		2nd cycle	7 (17.1)	6 (14.3)
	^a^*P* value	0.31	0.11		^a^*P* value	0.001	0.045
Headache	Control cycle	16 (39)	18 (42.9)	Nervousness	Control cycle	28 (68.3)	27 (64.3)
	1st cycle	17 (41.5)	17 (40.5)		1st cycle	19 (46.3)	23 (54.8)
	2nd cycle	8 (19.5)	19 (45.2)		2nd cycle	13 (31.7)	21 (50)
	^a^*P* value	0.02	0.65		^a^*P* value	< 0.001	0.13

^a^Cochran test

In addition to that, there was no statistically significant difference between the two groups in the amount of bleeding and AEs during the two intervention cycles.

### Safety assessments

There was no statistically significant difference between the two groups in side effects during the two intervention cycles. In the auriculotherapy group, five (12.2%) people in the first cycle and one (2.4%) person in the second cycle experienced redness, swelling, and pain at the site of the seeds. In the mefenamic acid group, three (7.1%) people in the first cycle and two (4.8%) people in the second cycle experienced stomach pain.

### Satisfaction rate

There was a statistically significant difference between the two groups in terms of satisfaction rate with the method in the first cycle (*P* = 0.01) and the second intervention (*P*<0.001). Satisfaction with auriculotherapy was 87.8% in the first cycle and 95.1% in the second cycle. While the satisfaction rate in mefenamic acid consumption was 64.3% in the first cycle and 57.1% in the second cycle.

## Discussion

The present study aimed to compare the effect of auriculotherapy and mefenamic acid on the severity and systemic symptoms of primary dysmenorrhea. The results indicated that the mean pain intensity in the auriculotherapy group using VAS in the first and second intervention cycles and VMS in the second cycle was significantly lower than the mefenamic acid group. Acupuncture is a branch of traditional Chinese medicine (TCM) with a mechanism similar to auriculotherapy. In a comparative study on the effect of acupuncture and mefenamic acid on PD, similar results to the present study indicated that the difference in mean pain intensity in the acupuncture group before and after the intervention was 3.51, which was higher than the mefenamic acid group [12]. In the aforementioned study, the difference in mean pain intensity was higher than our study, which could be related to performing intervention in three cycles compared to our study which was conducted in two cycles.

Aligned with the results of the present study, in Park’s study, individuals received auriculotherapy once a week for ten consecutive weeks, with the mean pain intensity difference of 2.5 before and after the intervention [17]. In our study, similar to Park’s study, the mean pain intensity difference in the auriculotherapy group before and after the intervention was 2.26.

Kim et al. conducted a study on the effect of ear acupuncture on dysmenorrhea in three groups. Group A on the first and second day of menstrual bleeding and group B once a week over 4 weeks, regardless of the menstrual bleeding time, received ear acupuncture for 15 min regularly. The seeds were inserted in 17 points. There was a significant difference in the rate of pain in group A compared to group B and the control group [21]. Compared with the aforementioned study, the decrease in mean pain intensity in our study was less than group A and the results were similar to group B, which may be due to the pressure of the number of more points a longer time.

The results of another study [5] also showed the effect of ear acupuncture on menstrual pain. In this study, seeds were planted in 6 points. The rate of pain reduction in this study was higher compared to our study, which could be due to the insertion of seeds in different points and more, or may performing auriculotherapy with more points in menstrual bleeding is more effective than its use during the menstrual cycle, even using various techniques of auriculotherapy may be effective in reducing pain.

With regard to systemic symptoms associated with PD, in our study, there was a significant decrease in the frequency of subjects with VMS grade 2 (few systemic symptoms) and 3 (common systemic symptoms) in the second cycle between the two groups auriculotherapy and mefenamic acid. In the auriculotherapy group in the second intervention cycle, none of the subjects had VMS grade 3. Considering the two options (yes/no) in systemic symptom assessment, there was a significant decrease in the frequency of symptoms headache, nausea, nervousness, fatigue, and diarrhea. Aligned with the results of our study, in a study conducted on the effect of acupressure in SP6 acupressure point on the systemic symptoms associated with dysmenorrhea, there was a significant decrease in symptoms fatigue, nausea, vomiting, headache, diarrhea, and change in the state of nervousness [22]. The results of another study entitled “the effectiveness of acupuncture in the treatment of nausea and vomiting in pregnancy” indicated that nausea and vomiting were decreased in the intervention group compared to the control group, although the difference was not statistically significant [23]. In our study, 53.7% of samples in the auriculotherapy group had nausea in the control cycle, which was significantly reduced to 34.1% and 19.5% in the first and the second intervention cycle, respectively. However, this difference was not significant in the mefenamic acid group. There was no significant difference in the frequency of vomiting between both groups. The results of the studies evaluating the effect of ear acupuncture in improving nausea, vomiting, and diarrhea in patients with gastric cancer [24], in the treatment of chemotherapy-induced nausea and vomiting in ovarian and endometrial cancer [25], and breast cancer [26], and the effect of electrical stimulation of the ear on the incidence of after cesarean section nausea and vomiting [27] have been in line with the results of the present study.

In the present study, 39% of subjects in the auriculotherapy group experienced headaches in the control cycle, which was significantly reduced to 19.5% in the second cycle, whereas in the mefenamic acid group, 42.9% of subjects experienced headaches in the control cycle which were increased by 45.2% in the second cycle, but it was not statistically significant. One of the mechanisms that can effectively improve menstrual headaches is the regulation of abnormal levels of prostaglandin F2α and plasma arginine vasopressin. Based on these findings, the effect of auriculotherapy on prostaglandin levels would reduce headaches [28].

With regard to the fatigue variable, there was no statistically significant difference between the two groups, but there was a significant decrease within each group. Auriculotherapy modulates excessive secretion of corticotropin-releasing hormone, cortisol hormone, and plasma adrenocorticotropic through the regulation of hypothalamic-adrenal axis function, which is expressed as one of the mechanisms by which auriculotherapy affects chronic fatigue syndrome [12]. Auriculotherapy can alter serum levels of anti-inflammatory biomarkers and induce reflexive reactions to relieve bodily harm [29]. Consistent with the results of our study in a study that conducted electro-acupuncture with ear seeds on patients with chronic fatigue syndrome, the results showed that fatigue was reduced [30]. Results from other studies also showed the effect of ear acupuncture in significantly reducing fatigue in patients with breast cancer [31] and fatigue after cesarean section [32].

In the present study, 68.3% of subjects in the auriculotherapy group experienced nervousness in the control cycle, which was significantly reduced to 46.3% and 31.7% in the first cycle and the second cycle, respectively. Aligned with the results of our study, ear acupuncture had significantly relaxed patients with anxiety disorders or major depressive disorder and considerably reduced anxiety, nervousness, and aggression [33]. Auriculotherapy can increase serotonin and endorphin levels [34]) and thereby helping to cope with negative emotions.

## Conclusion

The results showed that both auriculotherapy and mefenamic acid were effective treatments in reducing PD and its associated systemic symptoms, which was significantly higher in the auriculotherapy group. Since there was no follow-up cycle in our study, it is recommended to conduct studies with more auriculotherapy sessions accompanied by a follow-up cycle after the intervention. Considering the results of the present study, citing that no significant side effects were observed for auriculotherapy, this treatment can be used as a safe method, which also minimizes the side effect of the existing chemical treatments.

## Data Availability

The datasets used and analyzed during the current study are available from the corresponding author on reasonable request.

## Abbreviations

NSAIDs: Non-steroidal anti-inflammatory drugs
VAS: Visual analog scale
VMS: Verbal multidimensional scoring system
PD: Primary dysmenorrhea
TCM: Traditional Chinese Medicine
IRCT: Iranian Registry of Clinical Trials
CONSORT: Consolidated Standards of Reporting Trials
AEs: Adverse events
CRF: Case report form
